# Post-Translational Modifications of Deubiquitinating Enzymes: Expanding the Ubiquitin Code

**DOI:** 10.3389/fphar.2021.685011

**Published:** 2021-06-10

**Authors:** Yanfeng Wang, Feng Wang

**Affiliations:** Key Laboratory of Molecular Medicine and Biotherapy, School of Life Science, Beijing Institute of Technology, Beijing, China

**Keywords:** DUBs, post-translational modifications, phosphorylation, ubiquitination, sumoylation, acetylation, oxidation, hydroxylation

## Abstract

Post-translational modifications such as ubiquitination play important regulatory roles in several biological processes in eukaryotes. This process could be reversed by deubiquitinating enzymes (DUBs), which remove conjugated ubiquitin molecules from target substrates. Owing to their role as essential enzymes in regulating all ubiquitin-related processes, the abundance, localization, and catalytic activity of DUBs are tightly regulated. Dysregulation of DUBs can cause dramatic physiological consequences and a variety of disorders such as cancer, and neurodegenerative and inflammatory diseases. Multiple factors, such as transcription and translation of associated genes, and the presence of accessory domains, binding proteins, and inhibitors have been implicated in several aspects of DUB regulation. Beyond this level of regulation, emerging studies show that the function of DUBs can be regulated by a variety of post-translational modifications, which significantly affect the abundance, localization, and catalytic activity of DUBs. The most extensively studied post-translational modification of DUBs is phosphorylation. Besides phosphorylation, ubiquitination, SUMOylation, acetylation, oxidation, and hydroxylation are also reported in DUBs. In this review, we summarize the current knowledge on the regulatory effects of post-translational modifications of DUBs.

## Introduction

Ubiquitination is an essential post-translational protein modification mediated by the ubiquitin (Ub)-conjugating system, which is composed of a Ub-activating enzyme, E1, Ub-conjugating enzyme, E2, and Ub ligase, E3 (Hershko and Ciechanover, 1998). The human genome encodes more than 600 E3 ligases. The E2 enzyme is specific for this type of ligation and co-ordinately functions with multiple E3 ligases. Thus, the series of enzyme combinations determine the diversity of the ubiquitination process. Therefore, ubiquitination drives diverse biological signals that regulate the fate and function of a plethora of intracellular proteins (Reyes-Turcu et al., 2009).

Ubiquitination is a reversible process because the conjugated Ub molecule can be trimmed away from the target protein by deubiquitination enzymes (DUBs). The human genome encodes several kinds of DUBs, which can be divided into seven subfamilies (Nijman et al., 2005). Among these, six subfamilies include Ub-specific proteases (USPs), Ub C-terminal hydrolases (UCHs), ovarian tumour proteases (OTUs), Machado-Josephin domain-containing proteases (MJDs), MIU-containing novel DUB (MINDY), and zinc finger-containing ubiquitin peptidase 1 (ZUP1) (cysteine-dependent proteases). In contrast, the seventh family, Jab1/MPN domain-associated metallopeptidase (JAMM/MPN+), comprises of zinc-dependent metalloproteinases (Mizuno et al., 2007; Mevissen and Komander, 2017).

The human genome encodes a relatively small number of DUBs compared to Ub ligases; multiplicity does not seem to exist in DUBs. However, a large amount of regulatory mechanisms precisely expand the functions of DUBs in various Ub-related processes to ensure accurate biological responses (Sahtoe and Sixma, 2015). The regulatory mechanisms of DUBs can be globally classified into post-translational modifications (PTMs), substrate-induced changes, scaffold or binding protein-induced changes (Cannavo et al., 2007; Hsu et al., 2017), and inhibitor-induced changes (Nijman et al., 2005; Sahtoe and Sixma, 2015). The regulation of DUBs mainly involves control of catalytic activity, abundance, and localization of DUBs. Dysregulation of DUBs can cause a variety of disorders, such as cancer, and neurodegenerative and inflammatory diseases (Todi and Paulson, 2011; Lopez-Castejon and Edelmann, 2016; Pinto-Fernandez and Kessler, 2016).

**FIGURE 1:**
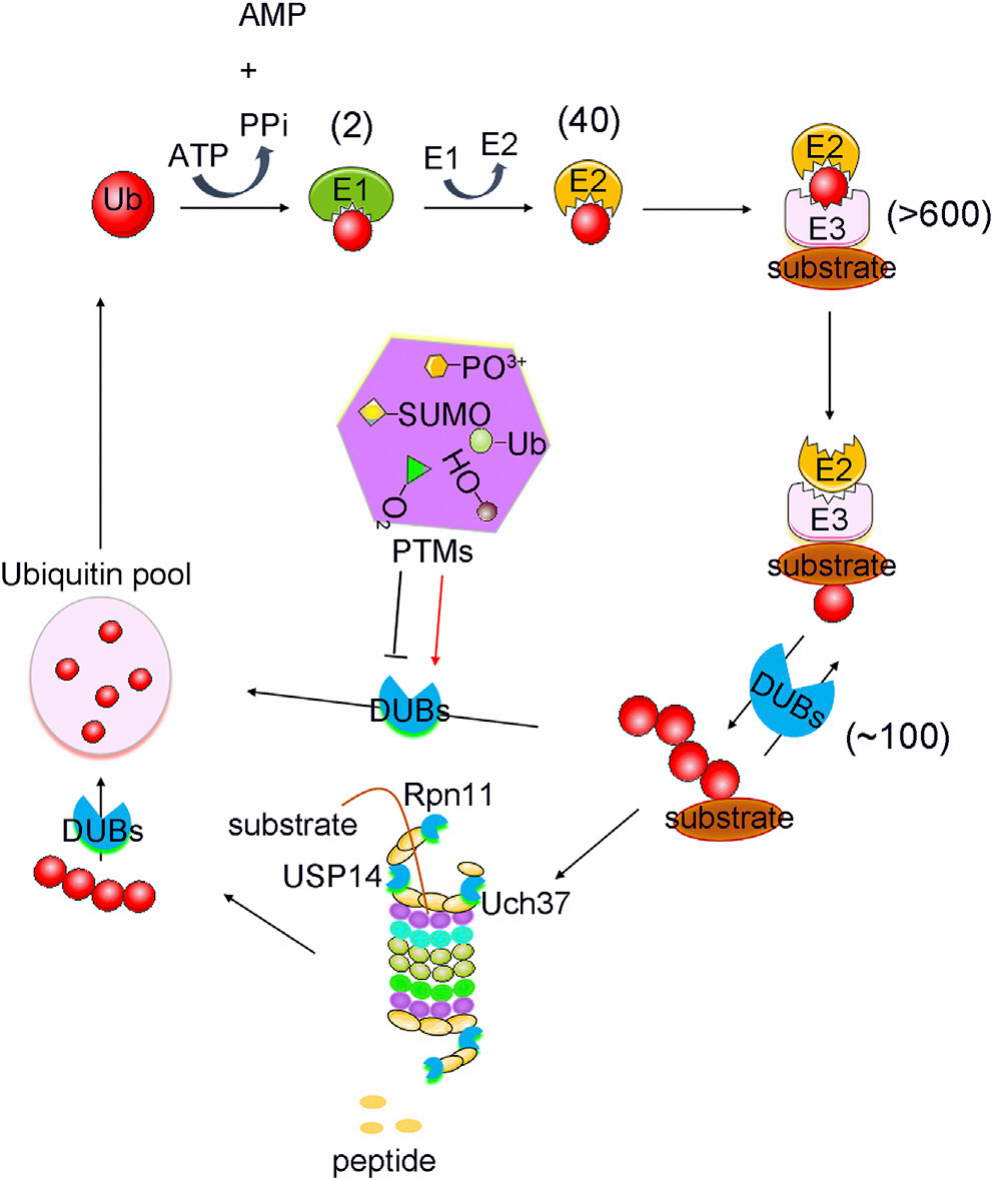
PTMs mediated ubiquitination and deubiquitination process. Protein Ubiquitination is a cascade reaction catalysed by E1 activating, E2 conjugating and E3 ligating enzymes. This can lead to the formation of distinct types of ubiquitin modifications, all of which can be reversed by deubiquitylating enzymes (DUBs). DUBs are regulated strictly by various post translational modifications (PTMs), including phosphorylation, ubiquitination, SUMOylation, acetylation, oxidation and hydroxylation. Numbers in brackets indicate the number of enzymes in each family.

With the advancement of analytical tools such as mass spectrometry, PTM sites of DUBs have been identified. Recently, the regulation of DUBs by PTMs and its physiological relevance have been revealed. Studies showed that PTMs can regulate the function of DUBs by altering factors such as its stability, localization, abundance, and catalytic activity, in addition to its involvement in the cell signalling pathway (Kessler and Edelmann, 2011). Here, we will review the regulatory effects of PTMs on DUBs, and its potential therapeutic role in tumour growth (Table 1). PTMs mainly consist of phosphorylation, ubiquitination, SUMOylation, acetylation, oxidation and hydroxylation, all of which have critical roles in the regulation of DUBs (Figure 1). Analysis of these regulatory processes may provide evidence for elucidating the function of DUBs and their potential as targets in novel therapeutic strategies.

**TABLE 1:** Summary of DUBs localization, PTMs and interaction profile.

DUB family	DUB (*H. sapiens*)	Localization	PTMs	Interactors	Correlation of PTMs with DUBs	References
UCHs	UCHL1	Endoplasmic reticulum membrane, lipid anchor, Cytoplasm	Ubiquitination, glycosylation, Oxidation, phosphorylation, Prenylation, Lipoprotein	Ubiquitin, SNCA, COPS5	Monoubiquitination of UCHL1 inhibit the binding of ubiquitin to UCHL1. Others were unknown	Das et al. (2006); Liu et al. (2009)
	UCHL3	Cytoplasm	Phosphorylation	Di-ubiquitin	Unknown	Dephoure et al. (2008); Misaghi et al. (2005)
	BAP1	Nucleus, Cytoplasm	Phosphorylation, ubiquitination	UBE2O, BRCA1, HCFC1, FOXK1, FOXK2	UBE2O interact with BAP1 and promote the ubiquitination of BAP1. Others were unknown	Okino et al. (2015)
OTUs	OTUB1	Cytoplasm	Phosphorylation, hydroxylation	UBE2N/UBC13, RNF128, USP8, FUS, ESR1	Hydroxylation of OTUB1 promote the interaction of OTUB1 with metabolism-associated proteins, such as UBE2N/UBC13. Others were unknown	Van Damme et al. (2012); Scholz et al. (2016)
	OTUD1	Unknown	Oxidation	SMURF1, IRF7	Unknown	Zhang et al. (2018)
	OTUD3	Cytosol, cytoplasm	Oxidation	NEDD4-1, RPF1	Unknown	Yuan et al. (2015); Zhou et al. (2013a)
	OTUD4	Nucleus, cytoplasm	Phosphorylation	K63-linked ubiquitin chain, MYD88, ALKBH3, USP7, USP9X	Phosphorylation of OTUD4 promote the binding and hydrolysis of OTUD4 to K63-linked ubiquitin chain. Others were unknown	Zhao et al. (2015); Zhao et al. (2018)
	OTUD5	Cytosol	Phosphorylation, oxidation	Ubiquitin, TRAF3	Phosphorylation of OTUD5 increased the recognition and binding of OTUD5 to ubiquitin. Others were known	Huang et al. (2012)
	OTULIN	Cytoplasm	Phosphorylation	RNF31, DVL2, β-catenin, LUBAC	Phosphorylation of OTULIN enhance the binding of OTULIN to β-catenin, while block the binding of OTULIN to LUBAC. Others were unknown	Damgaard et al. (2016); Keusekotten et al. (2013); Wang et al. (2020b)
	A20	Lysosome, nucleus, cytoplasm	Phosphorylation, ubiquitination	TNIP1, TAX1BP, TRAF2	Unknown	Song et al. (1996)
	Cezanne	Nucleus, cytoplasm	Hydroxylation	Ubiquitin, ZAP70, EGFR	Hydroxylation of cezanne inhibit the binding of ubiquitin to cezanne. Others were unknown	Pareja et al. (2012)
	Cezanne2	Nucleus,cytoplasm	Methylation, phosphorylation	TRAF6, UBC	Unknown	Xu et al. (2014)
MJDs	Ataxin3	Nucleus matrix, nucleus	Phosphorylation, ubiquitination, SUMOylation	Ubiquitin, CASP7, UBR2	Ubiquitination of ataxin3 enhance its binding to ubiquitin. Others were unknown	Scaglione et al. (2013); Weishäupl et al. (2019)
	JosD1	Cell membrane, cytoplasm	Ubiquitination	Ubiquitin, beta-actin/ACTB	Ubiquitination of JosD1 enhance its binding to ubiquitin. Others were unknown	Seki et al. (2013)
JAMMs	PSMD7	Cytosol, extracellular region or secreted, nucleus, proteasome complex, proteasome regulatory particle	Acetylation, ubiquitination	TRIM5, 26S proteasome	Unknown	Choudhary et al. (2009)
	PSMD14	Cytosol, extracellular region or secreted, nucleus, proteasome accessory complex, Proteasome complex	Phosphorylation	TXNL1	Unknown	Zhou et al. (2013a)
	EIF3H	Cytoplasm	Ubiquitination, phosphorylation	eIF-3, DHX33	Unknown	Zhang et al. (2015a)
	BRCC36	Cytoskeleton, nucleus, cytoplasm	Acetylation, phosphorylation	ABRAXAS1, BRCA1	Unknown	Zhou et al. (2013a)
	AMSH	Nucleus, early endosome, membrane, cytoplasm	Phosphorylation	SMURF2, RNF11	Unknown	Li and Seth. (2004)
	AMSH-LP	Cytosol, Endosome, Membrane	Acetylation, Phosphorylation	INCA1, RAB2A	Unknown	Van Damme et al. (2012)
	MPND	SWI/SNF complex	Acetylation, phosphorylation	E7	Unknown	Gaudet et al. (2011)
	PRPF8	Nucleus, nucleus speckle	Acetylation, methylation, phosphorylation	U5 snRNP, SNRNP40	Unknown	Bertram et al. (2017); Olsen et al. (2010)
USPs	USP1	Nucleus	Phosphorylation	UAF1, FANCD2, PCNA, WDR48	Phosphorylation of USP1 influence the interaction of USP1-UAF1 and promote the binding of USP1 to FANCD2 and PCNA.	Huang et al. (2006)
	USP4	Nucleus, cytoplasm	Phosphorylation, ubiquitination	CtIP/MRN, ADORA2A, RB1, USP15 or TβRI	Phosphorylation of USP4 promote its binding to USP15 and TβRI. Auto-deubiquitination of USP4 is required for USP4 to interact with CtIP/MRN.	Uras et al. (2012); Wijnhoven et al. (2015)
	USP6	Cell membrane, endosome, cytoplasm	Ubiquitination	Ca^2+^/Calmodulin, RAC1, CDC42	Ubiquitination of USP6 promote its binding to Ca^2+^/Calmodulin. Others were unknown	Shen et al. (2005a)
	USP7	Nucleus, PML body, cytoplasm, chromosome	Phosphorylation, oxidation, ubiquitination	FOXO4, MDM2	Phosphorylation of USP7 promote the stabilization of MDM2 through deubiquitinating it	Fernández-Montalván et al. (2007)
	USP8	Nucleus, endosome membrane, membrane protein, cell membrane, peripheral membrane protein, cytoplasm	Phosphorylation	14-3–3 protein, LC3, STAM2	Phosphorylation of USP8 promote its binding to 14-3–3 protein. Others were unknown	Dephoure et al. (2008); Row et al. (2009)
	USP9X	Cytoplasm, growth cone	Phosphorylation	ZAP70, SMAD4, DCX	Phosphorylation of USP9X promote the deuibiquitination of ZAP70. Others were unknown	Homan et al. (2014); Zhou et al. (2013b)
	USP10	Early endosome, Nucleus, cytoplasm	Phosphorylation	TRF6, p53, AMPK	Phosphorylation of USP10 promote the deuibiquitination and stabilization of p53 and AMPK.	Wang et al. (2015a); Yuan et al. (2010)
	USP11	Nucleus, cytoplasm, chromosome	Ubiquitination	NFKBIA, BRCA2	Unknown	Schoenfeld et al. (2004); Wiltshire et al. (2010)
	USP13	Cytosol, nucleoplasm	Phosphorylation	Aurora B, RAP80, c-Myc, SIAH2, BAG6	Phosphorylation of USP13 promote its interaction with aurora B, RAP80, and c-Myc. Others were unknown	Esposito et al. (2020); Scortegagna et al. (2011); Zhou et al. (2020)
	USP14	Cell membrane, peripheral membrane protein, cytoplasm	Phosphorylation	Ubiquitin, CXCR4, fANCC	Phosphorylation of USP14 promote its binding to ubiquitin. Others were unknown	Mines et al. (2009); Zhou et al. (2013a)
	USP15	Nucleus, mitochondrion, cytoplasm	Phosphorylation, ubiquitination	SMAD1, SMAD2	Auto-deubiquitination of USP15 promote its interaction with SMAD1	Cornelissen et al. (2014); Inui et al. (2011)
	USP19	Endoplasmic reticulum membrane, single-pass membrane protein	Oxidation	c-IAP1, c-IAP2, RNF123	Unknown	Mei et al. (2011)
	USP25	Cytoplasm, nucleus, cytoplasm	Phosphorylation, ubiquitination, SUMOylation	SYK, Sumo1, Sumo2, TRiC, ub chains	SYK-dependent phosphorylation of USP25 promote the stabilization of TRiC, SUMOylation of USP25 inhibit its binding to ub chains	Cholay et al. (2010); Denuc et al. (2009)
	USP28	Nucleoplasm	SUMOylation, oxidation	ZNF304, Fbw7	Unknown	Popov et al. (2007a); Popov et al. (2007b); Zhang et al. (2006)
	USP30	Mitochondrion outer membrane	Ubiquitination	EAP1, POMK	Unknown	Bingol et al. (2014); Huttlin et al. (2017)
	USP36	Nucleolus, cytoplasm	Phosphorylation	C-myc, NEDD4L	Unknown	Sun et al. (2015)
	USP37	Nucleoplasm, nucleus	Phosphorylation	FZR1/CDH1, CDT1	Phosphorylation of USP37 enhance its binding to the substrate adaptor CDH1. Others were unknown	Huang et al. (2011)
	USP39	Unknown	SUMOylation	Tri-snRNP, LRRK2	SUMOylation of USP39 promote its interaction with tri-snRNP.	Liu et al. (2015a)
	USP44	Nucleus	Phosphorylation, ubiquitination	CETN2, EZH2	Unknown	Lan et al. (2016); Suresh et al. (2010); Visconti et al. (2012)
	USP47	Cytoplasm	Acetylation, phosphorylation	BTRC, FBXW11, POLB	Unknown	Parsons et al. (2011); Peschiaroli et al. (2010)
	USP49	Nucleus	Phosphorylation	RUVBL1, PSMC5	Unknown	Zhang et al. (2013)
	CYLD	Cytoskeleton, centrosome, spindle cilium basal body, plasma membrane, cytoplasm perinuclear region	Phosphorylation, SUMOylation, oxidation	TRAF2, SPATA2, MAP3K7	Phosphorylation or SUMOylation of CYLD inhibit its interaction of TRAF2. Others were unknown	Eguether et al. (2014); ji et al. (2018); Schlicher et al. (2016)
	USPL1	Cajal body	Phosphorylation	ELL	Unknown	Hutten et al. (2014); Schulz et al. (2012)

## PTMs Regulate the Abundance of DUBs

The most straightforward mechanism affecting the biological function of a given protein is its intracellular concentration. Like other signalling proteins, this fact is also applicable to DUBs. The quantity of DUBs is cell or tissue-specific; UCHs subfamily of DUBs are highly abundant in neurons (Liu et al., 2009). In contrast, DUBs such as USP30 show relatively low expression levels in neurons. Therefore, this highlights the specific spatial and temporal functional of DUBs in different cells or tissues (Clague et al., 2015). In addition, the abundance of DUBs is strictly controlled by regulation of its transcription, translation, PTMs, and degradation. In the following sections, we summarize how PTMs such as phosphorylation and ubiquitination regulate the abundance of DUBs (Das et al., 2020).

### Regulation of DUB Stability and Abundance by Phosphorylation

Phosphorylation is an important kind of PTM that influences the essential physiological role of DUBs, and exerts its effect by elevating, stabilizing, or reducing its abundance. Phosphorylation can elevate DUB protein levels by altering their self-assembly and interaction with other partners. Results have shown that AKT-mediated phosphorylation of USP4 at the Ser445 residue is essential for it to form a complex with itself or with other protein partners, such as USP15 or TβRI (Zhang et al., 2012). In particular, when USP4 was co-expressed with USP15, an elevated USP4 protein level was detected. Additionally, AKT-induced phosphorylation of USP4 is required for maintaining the stability of USP4, thereby enhancing TGF-β-induced pro-tumorigenic responses in breast cancer cells. TGF-β-induced migration of MDA-MB-231 cells is inhibited by USP4 knockdown and PI (3) K–AKT signalling inhibitors, indicating that phosphorylation of USP4 plays a critical role in AKT-mediated breast cancer cell migration (Zhang et al., 2014; Wang W. et al., 2020). Therefore, phosphorylation is essential for stabilizing and maintaining the protein levels of USP4 and plays a potential role in breast cancer pathogenesis (Zhang et al., 2012).

Furthermore, protein kinase CK2-induced phosphorylation of USP7 at the Ser18 residue plays a major role in maintaining the stability of USP7 protein (Khoronenkova et al., 2012). Phosphorylation can prevent the ubiquitination of USP7 and prevent its degradation by the proteasome. The dephosphorylation of USP7 decreased its stability and makes it prone to proteasomal degradation. Generally, large amounts of USP7 are phosphorylated by CK2 and remain active in unstressed cells (Olsten and Litchfield, 2004). This promotes the deubiquitination and stabilization of Mdm2, which in turn leads to the degradation and downregulation of p53. However, when DNA damage occurs, USP7 is dephosphorylated by PPM1G, which induces p53 stabilization due to Mdm2 degradation, which suggests that inhibition of the phosphorylation of USP7, as a part of the DNA damage response (DDR), may exhibit a potential therapeutic effect (Fernández-Montalván et al., 2007; Khoronenkova et al., 2012; Pozhidaeva and Bezsonova, 2019).

On the contrary, phosphorylation can also reduce DUB protein levels by altering the degradation pathway of DUBs. For instance, SYK-dependent phosphorylation of USP25 at residue Tyr740 can sharply reduce its protein levels. This is not caused by proteasome-dependent degradation because addition of a proteasome inhibitor did not rescue USP25 proteins levels (Figure 3A). Therefore, it is reasonable to assume that SYK-dependent phosphorylation may activate other pathways, such as lysosomal degradation, to alter USP25 protein levels (Cholay et al., 2010; Kim et al., 2015).

### Ubiquitination Induces the Auto-Deubiquitination of DUBs

Ubiquitination and deubiquitination are two types of important PTMs. Usually, DUBs play a critical role in the Ub proteasome system (UPS) by deubiquitinating the protein substrate (Komander et al., 2009). Interestingly, DUB can also be ubiquitinated itself to alter its destiny either by promoting or decreasing its degradation. For example, many DUBs undergo mono/poly-ubiquitination modification processes, and are then subjected to proteasomal degradation, resulting in a decrease in DUB protein levels (Wada et al., 2006). However, several DUBs have an auto-deubiquitination mechanism to prevent its degradation. Studies have shown that USP4 is a stable DUB protein because it can deubiquitinate itself after being ubiquitinated by Ro52 (Wada and Kamitani, 2006; Zhang et al., 2012). Similar to that of USP4, the self-deubiquitination of USP25 confers a protection mechanism to prevent it from proteasomal degradation (Figure 3A) (Denuc et al., 2009).

## PTMs can Regulate the Localization of DUBs

The subcellular localization of DUBs is also a key factor in determining the function of DUBs (Clague et al., 2015). If both the enzyme and the substrate circulate freely, they will be diluted in the cytoplasm or separated into different subcellular organelles, which will not allow the enzyme-catalysed reaction to occur at an appropriate enzymatic rate. Currently, the localization of DUBs is garnering attention as a vital regulatory mechanism (Mevissen and Komander, 2017).

**FIGURE 2:**
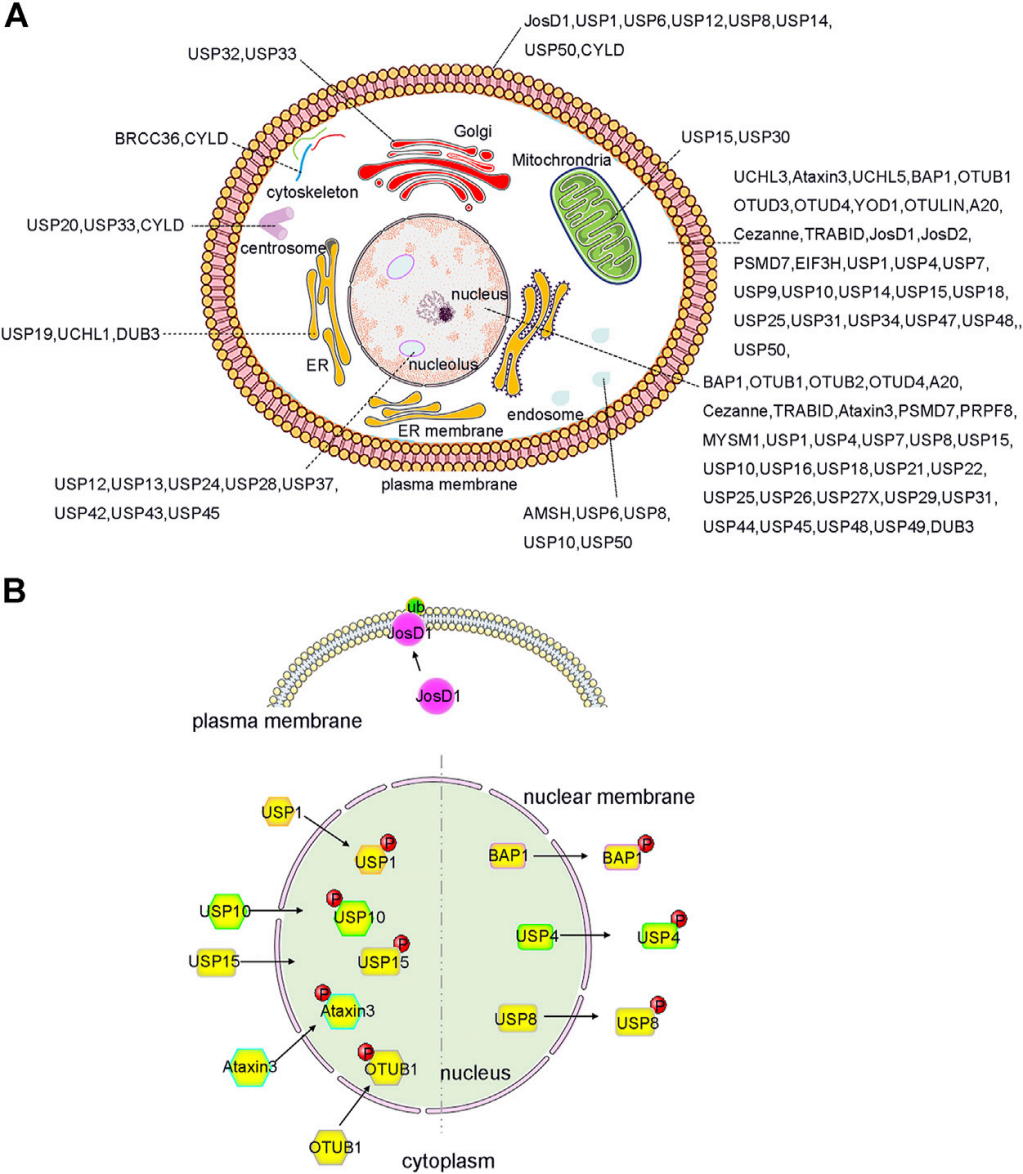
Localization and PTMs induced translocation of DUBs. **(A)** DUBs have been reported to localize and function in almost every intracellular compartment, such as nucleolus, cell membrane and mitochrondria etc., and have specific roles. Importantly, the function of DUB can be expanded by ensuring that a single DUB localizes to distinct organelles as shown, for example, for JosD1, USP4 and USP25 etc. **(B)** PTMs, such as phosphorylation and ubiquitination, play an important role in regulation the alternative localization of DUB. For example, phosphorylation of BAP1, USP4 and USP8 causes them to relocate from the nucleus to the cytosol. In contrast, phosphorylation of OTUB1, Ataxin3, USP15, USP10, and USP1 triggers its translocation from cytosol to the nucleus. Additionally, ubiquitination of JosD1 leads its translocation from cytosol to cell membrane. The figure was generated based on the reported studies.

Indeed, the subcellular localization of some DUBs is tightly controlled so as to facilitate its biological functions. In previous studies involving systematic large-scale proteomic analyses, the subcellular distribution of GFP-labelled DUBs was analysed in HeLa cell lines (Urbé et al., 2012) (Figure 2A). DUBs such as USP1, USP7, and USP11 were located in the nucleus, whereas other DUBs were localized in specific cellular compartments. Briefly, approximately nineteen DUBs were localized in the endoplasmic reticulum, twelve in the endosomes, eight in the mitochondria, and fifteen in the plasma membrane (Urbé et al., 2012; Clague et al., 2015). It is precisely because of these diverse localizations that the unique biological functions of DUBs are inferred. For example, the localization of USP36 in the nucleus allows it to specifically interact with the transcription factor c-Myc and deubiquitylate it (Sun et al., 2015). In the same way, USP30 located in the outer mitochondrial membrane can regulate mitochondrial morphology and plays a critical role in Parkin-mediated mitophagy (Nakamura and Hirose, 2008). Moreover, in lung cancer cells, the nucleus-localized USP15 can deubiquitylate histone H2B and inhibit degradation of the RE1-silencing transcription factor (REST) on the ribosome, which plays a pivotal role in cell cycle oscillations (Faronato et al., 2013).

More interestingly, recent studies have shown that the localization of DUBs in cells can be dynamically regulated to facilitate their complex biological functions (Kessler and Edelmann, 2011; Leznicki and Kulathu, 2017; Mevissen and Komander, 2017; Das et al., 2020). For example, PTMs can regulate and alter the localization of DUBs in a variety of ways, including PTM-induced DUB translocation from the cytoplasm to the nucleus, PTM-induced excretion of DUBs from the nucleus, and PTM-induced DUB translocation to the cell membrane (Figure 2B). All of these factors contribute to the functional diversity and substrate specificity of DUBs.

### Phosphorylation Regulates the Interactome and Localization of DUBs

Phosphorylation can also affect the function of various DUBs by altering its localization or interactome. Phosphorylation can cause some DUBs to be exported from the nucleus (Figure 2B). For instance, dephosphorylated USP4 accumulates in the nucleus, whereas the AKT-mediated phosphorylated form of USP4 (at residue Ser445) was primarily localized in the cytoplasm and cell membrane. The phosphorylation of USP4 prolongs its half-life on the plasma membrane, and activates TGF-β cell signalling through binding and deubiquitination of TGF-β receptors, which plays a crucial role in tumour cell migration and metastasis. This highlights the potential therapeutic role of USP4 phosphorylation in tumour progression (Zhang et al., 2012; Wang Y. et al., 2020). Recent studies have also shown the forms of USP15 phosphorylated at Thr149 and Thr219 residues are predominantly localized in the cytoplasm. In contrast, dephosphorylated USP15 relocates to the nucleus and plays an important role in spliceosome dynamics (Das et al., 2019).

Furthermore, phosphorylation can induce the translocation of specific DUBs into the nucleus (Figure 2B). For example, after DNA damage, Ataxia telangiectasia mutated (ATM)-induced phosphorylation of USP10 at residues Thr42 and Ser337 can promote the stability of USP10 and facilitate its translocation from the cytoplasm to the nucleus. Furthermore, phosphorylated USP10 can deubiquitinate and stabilize the tumour suppressor protein p53 by reversing its nuclear export and degradation *via* Mdm2. Therefore, phosphorylated USP10 can inhibit the growth of tumour cells without inducing mutations in p53, which implies that phosphorylation of USP10 has a potential therapeutic effect against tumours (Mueller et al., 2009; Yuan et al., 2010; Herhaus et al., 2015). Under oxidative stress, the phosphorylation of Ataxin3 at Ser111 is required for its nuclear localization (Costa and Paulson, 2012). Although progress has been made on the phosphorylation-induced localization changes of different DUBs, the understanding of the regulation mechanisms associated with PTMs, particularly in relation to DUB localization, is still limited.

Under certain conditions, phosphorylation-mediated changes in DUB localization can be observed by altering DUB interactions. Phosphorylation of USP1 on Ser313 can influence its interaction with the cofactor USP1-associated factor 1 (UAF1). The complex comprising of USP1 and UAF1 was localized in the cytoplasm. After phosphorylation of USP1, the complex translocated to the nucleus, where the recruitment of the Fanconi anemia protein FANCD2 and PCNA substrates mediated by a SUMO-like domain occurs in response to DNA damage (Huang et al., 2006; Garcia-Santisteban et al., 2012; Villamil et al., 2012; Olazabal-Herrero et al., 2015).

Similarly, DNA damage-induced ABL1/c-Abl (ABL proto-oncogene 1) activation can promote the phosphorylation of OTULIN at Tyr56, which enhances its interaction with β-catenin and blocks its binding to LUBAC (Keusekotten et al., 2013; Elliott et al., 2014; Schaeffer et al., 2014). Then, an increased OTULIN/β-catenin interaction promotes the stabilization of β-catenin and activation of Wnt/β-catenin signalling. This pathway plays a critical role in the progression and metastasis of triple-negative breast cancer (TNBC), metastasis, and resistance to cancer treatments. Therefore, targeting OTULIN or OTULIN phosphorylation may serve as an effective strategy for the treatment of patients with TNBC (Wang W. et al., 2020; Wang and Wu, 2020). Additionally, a study also reported that OTULIN is hyper-phosphorylated at Tyr56 residues during necroptosis, which can modulate ubiquitination of the receptor interacting protein kinase (RIPK1) and promote cell death (Douglas and Saleh, 2019).

**FIGURE 3:**
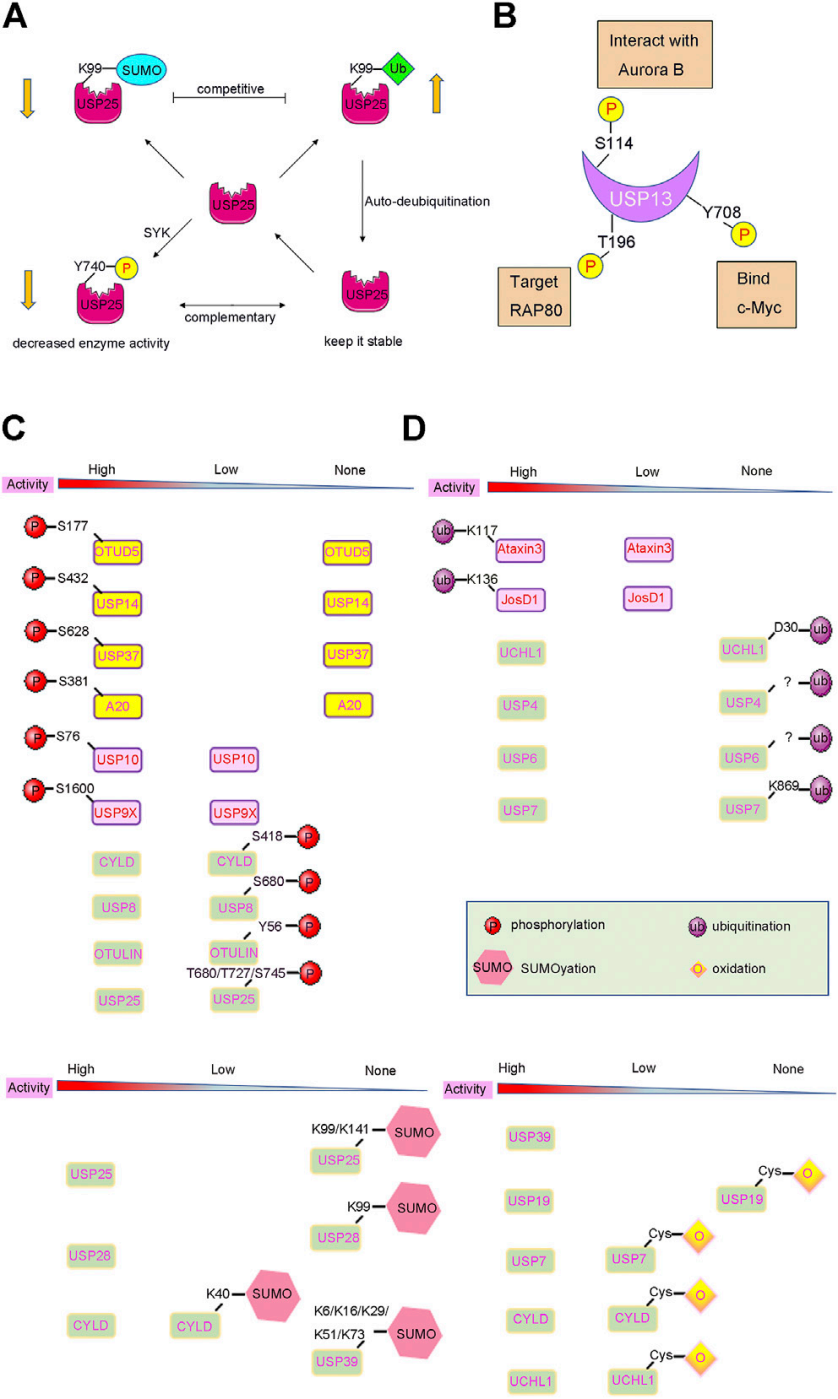
PTMs induced activity changes of DUBs. **(A)** Complex crosstalks of different PTMs often occur on DUBs with different effects. For example, USP25 can be modified by phosphorylation, SUMOylation and ubiquitination, which affect the activity and stability of USP25. **(B)** Many DUBs undergo one or more PTMs, such as USP13 can be phosphorylated at Ser114, Thr196, Tyr708, leading to different biological events. **(C)** and **(D)** DUB activities can also be changed by PTMs such as phosphorylation, ubiquitylation, and SUMOylation. The arrow below shows the change in DUBs’ enzyme activity after PTMs modification.

Furthermore, the kinase Aurora B plays an important role in mitosis. The protein level of Aurora B varies during the course of cell division. The abnormal regulation of Aurora B during interphase leads to cell cycle defects, which are usually associated with aberrant chromosomal condensation and segregation (Gully et al., 2012). Studies have shown that Aurora B phosphorylates USP13 at Ser114 and promotes its interaction with USP13. USP13, in turn, deubiquitinates Aurora B to protect it from proteasomal degradation, thereby stabilizing the protein levels of Aurora B (Figure 3B). Therefore, this ensures proper regulation of Aurora B and consequently the cell cycle, thereby preventing several human cancers, especially those cancers where Aurora B overexpression has been reported, such as ovarian, lung, brain, and skin-melanoma related cancers (Zeng et al., 2007; Chen et al., 2009; Esposito et al., 2020).

The receptor-associated protein 80 (RAP80) can recruit BRCA1 to DNA double-strand breaks and induce a DDR (Kim et al., 2007; Sobhian et al., 2007; Hu et al., 2011). Recent studies have reported that the deubiquitinating enzyme USP13 can regulate DDR by targeting RAP80. Mechanistically, USP13 is phosphorylated at Thr196 by ATM following DNA damage, which facilitates the localization of USP13 at the double-strand breaks. Then, USP13 can deubiquitinate RAP80 and stimulate its recruitment to induce an adequate DNA DDR (Figure 3B). Overall, USP13 functions as an essential regulator of DNA repair, and plays a vital role in the resistance of ovarian cancer cells to chemotherapy, and may provide a new approach for the treatment of ovarian cancer (Li et al., 2017).

Moreover, a recent study also found that CDC-like kinase 3 (CLK3) or the cholangiocarcinoma-associated CLK3-Q607R mutant can directly phosphorylate USP13 at Tyr708, and promote its binding to c-Myc (Nayler et al., 1997; Zhou et al., 2020) (Figure 3B). Therefore, this stabilizes c-Myc and activates the transcription of genes related to purine metabolism (Figure 3B). Thus, CLK3-mediated phosphorylation of USP13 at Tyr708 promotes cholangiocarcinoma progression by activating c-Myc-induced purine synthesis, providing a new and viable therapeutic target for the treatment of cholangiocarcinoma associated with CLK3 mutations (Zhou et al., 2020).

### Ubiquitylation Induces Changes in the Localization and Interactome of DUBs

Ubiquitination plays an essential role in numerous cellular processes, such as protein degradation, cell cycle, and transcriptional regulation (Reyes-Turcu et al., 2009). Similar to protein phosphorylation, ubiquitination is a critical PTM occurring in DUBs, which can alter the localization of DUBs and regulate their physiological functions (Leznicki and Kulathu, 2017; Das et al., 2020).

Josephin domain containing 1 (JosD1), a DUB of the MJD subfamily, mainly associates with the cytoskeleton. However, when ubiquitination occurs on JosD1, it tends to localize on the cell membrane (Figure 2B) and plays a vital role in membrane dynamics, cell motility, and endocytosis (Seki et al., 2013; Zeng et al., 2020).

Furthermore, BAP1, a member of the UCHs subfamily, is predominantly located in the nucleus and functions as a tumour suppressor (Cheung and Testa, 2017; Lee et al., 2020; Han et al., 2021). The lysine residues near the nuclear localization sequence (NLS) of BAP1 can be ubiquitinated, which leads to the accumulation of ubiquitinated BAP1 in the cytosol. Moreover, when BAP1 is co-expressed with UBE2O, it displays significant cytoplasmic staining. This is also due to the ubiquitination of BAP1 near the NLS by UBE2O. Studies have shown that the auto-deubiquitination of BAP1 can counteract this process through intramolecular interactions, thereby ensuring its function in tumour suppression (Mashtalir et al., 2014; Okino et al., 2015).

Similar to phosphorylation of DUBs, ubiquitination can also change the localization of DUBs, which can be detected by changing the interaction partners of DUBs. However, auto-deubiquitination, which is dependent on the catalytic activity of DUBs, can counteract the effect of ubiquitination modification and promote the interaction between DUB and its substrates. For instance, USP4 can be ubiquitinated at multiple sites such as the cysteine residues. Auto-deubiquitination can also occur at these sites, which is required for USP4 to interact with CtIP/MRN and promote DNA repair (Wada and Kamitani, 2006; Liu H. et al., 2015; Wijnhoven et al., 2015). Moreover, there are other DUBs such as USP11 and USP15 that also undergo ubiquitination modification and alter their interaction with other proteins. This process can be counteracted by auto-deubiquitination. Studies have shown that the substrate SMAD2/3 can interact with WT USP15, and not with a catalytically dead USP15 mutant, implying that there may be an auto-deubiquitination-dependent interaction as well (Inui et al., 2011; Wijnhoven et al., 2015). There are many other DUBs including USP18, USP21, USP30, USP44, USP47, USP, PSMD7, PSMD14, AMSH, PRPF8, USPL1 etc which are tightly regulated by interactors (Choudhary et al., 2009; Peschiaroli et al., 2010; Gaudet et al., 2011; Parsons et al., 2011; Zhang et al., 2013; Hutten et al., 2014; Huttlin et al., 2017), but it’s not clear whether the PTMs occurring on DUBs and affecting their interactors (Li and Seth, 2004; Malakhova et al., 2006; Olsen et al., 2010; Suresh et al., 2010; Visconti et al., 2012; Khan et al., 2015; Lan et al., 2016).

### Hydroxylation Alters the Localization of DUBs

Hypoxia is a commonly encountered physiological stress that can induce an active response in mammalian cells through a transcription factor named hypoxia-inducible factor (HIF) (Schofield and Ratcliffe, 2004; Semenza, 2009). Oxygen-dependent hydroxylation (-OH) is also a functional PTM that can impact the localization of DUBs by altering their interactome (Sowa et al., 2009; Scholz et al., 2013). For example, when OTUB1 is hydroxylated at the Asn22 residue by factor inhibiting HIF (FIH), the interactome and substrates of OTUB1 are elevated, particularly its interaction with metabolism-associated proteins. This suggests that OTUB1 may function as a possible link between oxygen sensing and metabolic regulation. In addition, the protein stability of OTUB1 is not changed by the hydroxylation of Asn22 (Scholz et al., 2016; Van Damme et al., 2012). Recent studies have identified Cezanne as a novel substrate of the asparaginyl β-hydroxylase FIH1. Cezanne is hydroxylated at Asn35 of the UBA domain in an oxygen- or FIH1-dependent manner, which inhibits the binding of Ub to Cezanne (Mader et al., 2020).

## PTMs can Regulate the Specificity and Activity of DUBs

DUBs are active and substrate-specific enzymes. However, certain DUBs require Ub-binding or modulation to form their active conformation. In a cellular environment, DUB activity is tightly regulated because uncontrolled activation can be detrimental for cells (Reyes-Turcu et al., 2009; Li and Reverter, 2021). Herein, PTMs are a critical approach to regulate the activity and specificity of DUBs, and also play an important role in DUBs-related diseases.

### Phosphorylation Induces DUB Activity and Specificity

Phosphorylation is a central PTM that can regulate the function of a variety of DUBs by directly influencing its catalytic activity (López-Otín and Hunter, 2010). Phosphorylation of DUBs has been shown to modulate, activate, and inhibit the activity of various DUBs (Figure 3C).

**FIGURE 4:**
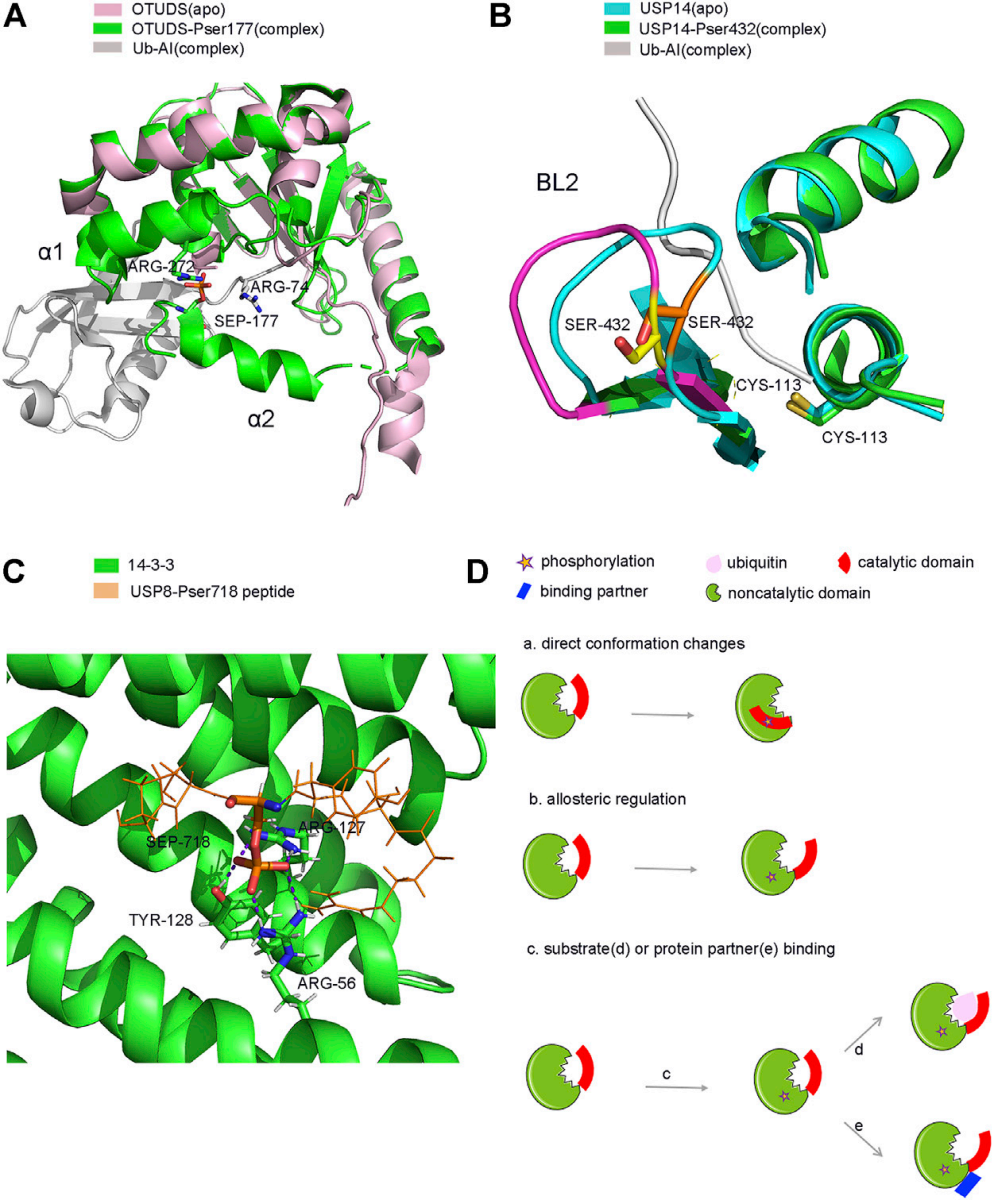
Structure and model of PTMs-induced conformation changes of DUBs. **(A)**. Phosphorylation induced conformation changes of OTUD5 after Ub binding. **(B)**. Phosphorylation induced direct conformation changes of USP14 **(C)**. Phosphorylation induced partner binding of USP8. **(D)**. Model of PTMs induced conformation changes of DUBs. **(a)**. phosphorylation-induced conformation changes **(b)**. phosphorylation-induced allosterically regulated conformation changes **(c)**. phosphorylation-induced substrate **(d)** or protein partner **(e)** binding and conformation changes.

Firstly, phosphorylation can activate or enhance the activity of several DUBs. OTUD5 is a relatively well studied protease, and phosphorylation is known to activate its catalytic activity (Figure 3C). OTUD5 purified from *E. coli* is inactive, but can be activated when the Ser177 residue of OTUD5 is phosphorylated by CKII instead of by mimic phosphorylation (Dephoure et al., 2008; Mayya et al., 2009). Phosphorylation alone does not alter the structure of OTUD5, however, its structure changes significantly upon Ub binding. Therefore, phosphorylation is vital for the recognition of Ub by OTUD5. Structural studies have shown that the phosphate group can form a salt bridge with the distal end of the Ub substrate and the highly inwardly oriented α6 fragment of the OUT domain (Figure 4A). This pattern of substrate recognition is unique among DUBs containing PTMs with known structures (Komander, 2010; Huang et al., 2012). Interestingly, phosphorylation-driven conformational changes are a typical feature of protein kinase activation (Taylor and Kornev, 2011). OTUD5 activation is similar to protein kinases. Therefore, OTUD5 is an archetype that establishes a new connection between two critical post-translational modifications, phosphorylation and ubiquitination. The catalytic activity of DUB depends entirely on the phosphorylation of a single suitable site (Huang et al., 2012).

USP14, a DUB that reversibly binds to the proteasome, can negatively regulate the proteasome by trimming the Ub chain on the proteasome-bound substrate. Purified and recombinant USP14 is inactive and is largely activated when bound to the proteasome (Borodovsky et al., 2001; Koulich et al., 2008; Lee et al., 2010). However, a study found that proteasome-free forms of USP14 do exist in the cell, and their physiological functions remain unknown (Zhou H. et al., 2013). Until 2015, studies showed that AKT-induced phosphorylation of USP14 at residue Ser432 or a mimetic phosphorylation at Ser432 can significantly promote the activity of USP14 (Figure 3C). It was able to hydrolyse K48, K63 di-Ub, Ub-AMC, but not linear di-Ub (Di-Ub). Simultaneously, Ser432 phosphorylation or binding to the proteasome can synergistically increase the activity of USP14, suggesting that there are two different mechanisms to regulate the activity of USP14 (Xu et al., 2015). Therefore, upon phosphorylation at Ser432, USP14 can release self-inhibition and promote hydrolysis of the Ub substrates (Figure 4B). It is different from the mechanism by which the proteasome activates USP14.

Furthermore, phosphorylation of USP37 is a cell cycle-dependent event. It is induced in the G1 phase, peaks in the G1/S phase, and is no longer phosphorylated in the later stages of mitosis. Thus, USP37 plays an important role in cell cycle regulation (Huang et al., 2011). USP37 binds to the substrate adaptor CDH1 and removes the polyubiquitin chain, which is the degradation signal, from cyclin A (Lukas et al., 1999). The USP37 activity maximizes when Ser628 of USP37 is phosphorylated by CDK2 (Huang et al., 2011) (Figure 3C). One possible mechanism is that phosphorylation can enhance the binding of USP37 to the substrate by altering the conformation of its Ub interaction motif. However, how Ser628 phosphorylation promotes the activity of USP37 remains unknown. A detailed structural analysis of phosphorylated USP37 may be required to reveal this mechanism.

Studies have also reported that phosphorylation of A20 could promote the cleavage of K63-linked polyubiquitin chains by its OTU domain. Results showed that recombinant A20 purified from *E. coli* failed to cleave K63-linked tetraubiquitin (Komander and Barford, 2008), whereas A20 purified from mammalian cells cleaved the K63-polyubiquitin chain. In fact, IκKβ-mediated phosphorylation of A20 at the Ser381 residue plays an essential role in facilitating cleavage of K63-linked polyubiquitin chains by A20 (Figure 3C). This also clarifies the molecular mechanism of A20 in suppressing inflammation-associated signalling pathways (Lin et al., 2008; Wertz et al., 2015).

Similarly, USP10 purified from *E. coli* exhibits a defective deubiquitinase activity. The deubiquitinase activity of USP10 was significantly increased after USP10 was phosphorylated at the Ser76 residue by AMPK under energy stress (Figure 3C). Subsequently, it deubiquitinates AMPK and facilitates the further activation of AMPK, forming a feedforward loop (Hardie, 2011; Hornbeck et al., 2015). The phosphorylated Ser76 site lies in a predicted unstructured region external to the catalytic UCH domain of USP10 (Bhattacharya et al., 2020). Phosphorylation may promote the activity of USP10 by inducing conformational changes in USP10 or affecting the recognition and binding of USP10 to Ub substrates. In this context, the detailed structure of the phosphorylated form of USP10 is important for elucidating the essential role of phosphorylation in modulating the catalytic activity of USP10 (Deng et al., 2016).

TCR-dependent phosphorylation at residues Ser1600 of USP9X enhances its catalytic activity (Figure 3C) and makes it competent to deubiquitinate ZAP70 (LoGrasso et al., 1996; Mayya et al., 2009; Mayya and Han, 2009; Naik et al., 2014). Ser1600 lies within the UCH hydrolase domain of USP9X, and phosphorylation at Ser1600 confers USP9X with an increased catalytic activity (Paudel et al., 2019). USP9X functions as a positive regulatory switch during T lymphocyte activation by eliminating the monoubiquitination inhibition of ZAP70 (Naik and Dixit, 2016).

In contrast, phosphorylation can also exhibit an inhibitory effect on the catalytic activity of DUBs, as demonstrated in CYLD. CYLD, a tumour suppressor (Massoumi, 2011), can remove K63-linked Ub chains from a large number of inflammation-related substrates, such as TRAF2 and TRAF6, and inhibit inflammatory signalling and cell cycle progression by inactivating these substrates (Brummelkamp et al., 2003; Kovalenko et al., 2003). IKKε-induced phosphorylation at Ser418 of CYLD decreased its activity (Figure 3C) and completely blocked CYLD-mediated deubiquitination of TRAF2, thereby promoting tumorigenesis in breast cancer cells (Hutti et al., 2009; Massoumi, 2011; Eguether et al., 2014).

Compared to CYLD, VRK2 kinase-mediated phosphorylation of USP25 at residues Thr680, Thr727, and Ser745 also suppresses the deubiquitinating activity of USP25 (Kim et al., 2015) (Figure 3A). Impaired USP25 destabilizes the molecular chaperone TRiC protein, which is responsible for monitoring protein misfolding. TRiC functions as the first line of defense to prevent misfolded protein aggregation in neurodegenerative diseases (Tam et al., 2006; Kim et al., 2014). This indicates that inhibition of USP25 phosphorylation may have a potential role in the treatment of neurodegenerative diseases (Kim et al., 2015).

Additionally, aggregation of Ataxin3 can cause dendritic and synaptic loss in cultured neurons, and is involved in spinocerebellar ataxia type 3, also known as Machado-Joseph disease (Kawaguchi et al., 1994). A study demonstrated that glycogen synthase kinase 3β (GSK 3β)-induced phosphorylation of Ataxin3 at Ser256 can regulate the aggregation of Ataxin3 (Fei et al., 2007). The phosphorylation of Ataxin3 at the Ser12 residue adjacent to the catalytic domain can also counter the neuromorphological defects caused by the decrease in deubiquitinase activity. Additionally, mutations in the Ser12 residue can reduce protein aggregation, and neuronal and synapse loss, and are implicated in neurodegenerative diseases (Matos et al., 2016).

UCHL3 is phosphorylated at Ser75 and activated by ATM upon DNA damage. In turn, UCHL3 deubiquitinates RAD51 and promotes the binding between BRCA2 and RAD51, which play an important role in DNA damage repair and the resistance of cancer cells to radiation and chemotherapy (Davies et al., 2001; Luo et al., 2016; Zhao et al., 2017). Therefore, inhibition of UCHL3 activity or UCHL3 phosphorylation may provide new insights in cancer therapy. Obviously, the biological role and mechanism of UCHL3 phosphorylation has not been fully understood yet, and this requires further research (Misaghi et al., 2005).

Phosphorylation can inhibit the activity of USP8 in a cell cycle-dependent manner. In the interphase stage of cell division, the Ser680 residue of USP8 is phosphorylated, which enables USP8 to bind to the 14-3-3 protein This binding in turn inhibits the catalytic activity of USP8 (Mizuno et al., 2007) (Figure 3C). Meanwhile, in the M phase, the dephosphorylation of USP8 at Ser680 can enhance its catalytic activity (Mukai et al., 2008; Pohl and Jentsch, 2008). Moreover, a mutation in the Ser680 residue can also enhance the activity of USP8, including hydrolysis of K48 or K63 polyubiquitin chains and intracellular substrates both *in vivo* and *in vitro*. It is plausible that after the 14-3-3 protein allosterically binds to phosphorylated USP8; USP8 undergoes a conformational change from an active state to an inhibitory state (Centorrino et al., 2018). It is also possible that the binding of the 14-3-3 protein covers the catalytic domain of USP8 and prevents the substrate from entering the catalytic centre of the enzyme. Moreover, the binding site of the 14-3-3 protein on USP8 is very close to the catalytic active centre of USP8, which is only about 60 amino acids apart (Mizuno et al., 2007; Ernst et al., 2013). Additionally, EGFR kinase-induced phosphorylation of USP8 at Tyr717 and Tyr810 residues elevates its activity and activates the EGF receptor kinase-mediated inhibition of ciliogenesis. This process plays a critical role in the regulation of ciliogenesis in dividing cells (Row et al., 2009; Kasahara et al., 2018). Therefore, further structural studies may provide us with new insights into the molecular mechanism of phosphorylation-dependent alterations in USP8 (Figure 4C).

Interestingly, phosphorylation can alter the specificity of DUBs toward hydrolysis of Ub chains. A study found that OTUD4 purified from *E. coli* tends to hydrolyse K48-linked Ub chains, whereas OTUD4 purified from HEK293T cells preferentially hydrolyse K63-linked Ub chains (Mevissen et al., 2013; Zhao et al., 2015). Studies have confirmed that casein kinase II (CK2) induces the phosphorylation of OTUD4 at residues Ser202, which is adjacent to the catalytic OTU domain (Olsten and Litchfield, 2004). Therefore, phosphorylation alters the tendency of OTUD4 to hydrolyse Ub chains from that of K48- to K63-linked Ub chains. This process plays an essential role in innate immune signalling pathways (Zhao et al., 2018).

Therefore, PTMs such as phosphorylation can alter the activity of DUBs and its specificity towards Ub linkage types (Table 2). This is an exciting new concept that may be widely adopted in the future. Furthermore, it will provide a unique perspective for understanding how DUBs regulate different physiological processes through PTMs such as phosphorylation. Ubiquitylation can both positively or negatively regulate DUBs activity.

**TABLE 2:** Summary of phosphorylation induced DUBs’ activity change.

DUBs	Kinase	Phosphorylation site	Feature	Structure	Physiology	References
OTUD4	CK2(casein kinase II)	Ser202/Ser204 (adjacent to the OTU domain, and mimetic phosphorylation can mildly promote the DUB activity)	Convert to hydrolysis K63 from K48	No structure	Regulate MyD88-dependent NF-κB signaling	Zhao et al. (2018)
OTUD5	CK2(casein kinase II)	Ser177 (lies in an unstructured region of the protein immediately preceding the predicted core OTU domain)	pSer177- OTUD5 showed robust activity against K48/K63 di-ub and good activity against K11-linked substrate, while linear di-ub was not processed	Crystal (pSer177 does not affect the structure of apo OTUD5; phosphorylated loop folds over the ub-al C terminus to stabilize enzyme and exclude water from the active site	A regulator of innate immunity	Huang et al. (2012)
OTULIN	ABL1 (ABL proto-oncogene 1)	Tyr56 (adjacent to the OTU domain)	Promote the interaction of OTULIN/β-catenin and the activation of wnt/β-catenin signaling	Crystal structure of OUT domain	Playing a critical role in the triple-negative breast cancer progression and metastasis	Keusekotten et al. (2013); Wang et al. (2020a)
A20	IκKβ	Ser381, Ser480, Ser565, Thr625 (substitution of all four residues alone attenuated cleavage of K63-linked tetraubiquitin)	Hydrolysis K48- or K63-linked tetraubiquitin but not linear tetraubiquitin	Crystal	A20 phosphorylation suppresses	Wertz et al. (2015)
				FL OTU	Inflammatory signaling	
USP14	Akt	Ser432 (within a catalytic domain, located within BL2, shifts its position over a distance of 3–5 Å in pSer432 form compared with inactive free form, and an adduct between Ubal-USP14 and S432E mimetic also promote USP14 activity)	PSer432 and S432E mimetic all promote K48/K63 di-ub or Ub-AMC deubiquitination activity, while linear di-ub was not cleaved	Crystal	S432 phosphorylation and interaction with proteasome maybe	Xu et al. (2015)
				USP domain	Two different regulatory mechanisms for USP14	
USP37	CDK2 in G1/S cell cycle	Ser682(within a catalytic domain)	USP37 phosphorylation correlated with its cell cycle-specific DUB activity	No structure	Cell cycle	Huang et al. (2011)
USP8	Unknown		USP8 is catalytically inhibited in a phosphorylation-dependent manner by 14-3-3s protein binding during the interphase, while the activity is enhanced in the M phase where usp8 was dephosphorylated	Crystal structure with USP8 specific inhibitor	Cell cycle	Mizuno et al. (2007)
USP15	CDKs	Thr149/Thr219 (located at the UBL domain, two sites are in the linker of DUSP and UBL domain, affects the interaction of USP15 to other protein)	Regulate localization and interaction with SART3 protein and spliceosome deubiquitination	Crystal structure of SART3-USP15DUSP-UBL	Regulate spliceosome dynamics	Das et al. (2019)
USP10	AMPK	Ser76	Remove Lys63-linked polyubiquitin from the activation loop of AMPK	Unknown structure	Energy stress	Deng et al. (2016)
USP13	Aurora B	Ser114	USP13 controls aurora B stability *via* enzymatically independent mechanisms	Unknown structure	Cell cycle	Esposito et al. (2020)
	ATM	Thr196	USP13 regulates DNA damage repair by targeting RAP80	Unknown structure	DNA repair	Li et al. (2017)
	CLK3	Tyr708	Enhancing c-Myc stabilization	Unknown structure	Cholangiocarcinoma progression	Zhou et al. (2020)
USP4	AKT	Ser445(located at the minimal catalytic domain USP4D1D2)	Redirect USP4 subcellular localization to the plasma membrane	Crystal structure	TGF-β signaling, cancer	Zhang et al. (2012)
USP7	CK2	Ser18	Required for the stability of USP7	Crystal structure	DNA damage repair	Khoronenkova et al. (2012)
USP28	ATM	Ser67/Ser714	Stabilize DNA damage signaling protein	Crystal structure	DNA damage repair	Zhang et al. (2006)
USP25	SYK tyrosine kinase	Thr680, Thr727, Ser745 (adjacent to the catalytic domain)	Suppress the deubiquitination activity	Crystal structure	Protein stability	Kim et al. (2015)
UCHL3	ATM	Ser75	Regulate BRCA2-RAD51 pathway	Crystal structure	DNA damage repair	Luo et al. (2016)
CYLD	IKKε	Ser418	Inhibit the catalytic activity of CYLD	Crystal structure	Oncogenic transformation	Hutti et al. (2009)

### Ubiquitylation

DUBs can cleave the Ub chains on substrates and play an essential role in many cellular processes. Interestingly, DUBs themselves can also be subjected to ubiquitination modifications. This type of modification is emerging as a critical regulator of DUB function as it exhibits both negative and positive regulatory effects.

Studies have shown that ubiquitination can positively regulate the activity of DUBs, such as that observed in the MJD subfamily of DUBs. Ataxin3 is a member of the MJD subfamily and is a DUB implicated in protein quality control. The activity of Ataxin3 is closely related to neurodegenerative disorders (Kawaguchi et al., 1994; Stevanin et al., 1995; Weishaupl et al., 2019). The ubiquitination of Lys117 near the catalytic groove of Ataxin3 can enhance its Ub chain cleavage activity (Figure 3D), but does not alter its preference for K63-linked Ub chains (Todi et al., 2010; Todi et al., 2009). JosD1, another member of the MJD subfamily, exhibits limited cleavage activity toward K48- or K63-linked Ub chains. In contrast, the ubiquitinated forms of JosD1 at the Lys136 residue exhibit enhanced cleavage activity of Ub chains (Figure 3D). This result indicates ubiquitination can positively regulate the activity of JosD1, and that JosD1 plays an important role in membrane dynamics (Seki et al., 2013).

Moreover, ubiquitination of DUBs can also negatively regulate their activity. UCHL1, a member of the UCH subfamily of DUBs, is highly expressed in neurons, and is involved in several neurodegenerative diseases, including Alzheimer’s and Parkinson’s disease (Bower et al., 1999; Leroy et al., 1998). Studies have shown that the monoubiquitination of residues near the active site of UCHL1 can restrict its enzymatic activity (Figure 3D) by inhibiting the binding of Ub to UCHL1. Therefore, the permanent monoubiquitination status of UCHL1 prevents its deubiquitinase activity and reduces Ub levels in cells. Interestingly, UCHL1 can intramolecularly modulate its monoubiquitination status, which depends on its hydrolytic activity (Osaka et al., 2003; Meray and Lansbury, 2007).

Interestingly, auto-deubiquitination can occur on some DUBs after they are ubiquitinated. USP6, a member of the USP subfamily, was initially identified as an oncogene (Madan et al., 2016; Nakamura et al., 1992). A study demonstrated that USP6 is mono- or poly-ubiquitinated, which promotes its auto-deubiquitination through direct binding to Ca^2+^/Calmodulin (Shen C. et al., 2005). USP4 was first identified as a protein with a high sequence similarity to USP6 and has been thought to function as a DUB (Gupta et al., 1993). Studies have shown that wild-type USP4 is mono- or poly-ubiquitinated by Ro52. The ubiquitination level of a mutated form of USP4 (at the active site Cys311) was considerably elevated (Wada and Kamitani, 2006). These results indicated that self-deubiquitination occurred in wild-type USP4, and the mutation may inhibit the self-deubiquitination activity of USP4 (Wada and Kamitani, 2006). Simultaneously, a study showed that USP7 can also be ubiquitinated at the Lys869 residue (Figure 3D), but its function is unclear (Fernández-Montalván et al., 2007).

### SUMOylation Inhibits the Activity of DUBs

SUMOylation is also an essential and reversible PTM with significant roles in various cellular processes (Geiss-Friedlander and Melchior, 2007). Similar to ubiquitination, SUMOylation is a process involving the conjugation of a small peptide SUMO to the substrates *via* a hierarchical interplay of three enzymes: SUMO activating enzyme E1, SUMO-conjugation enzyme E2, and SUMO-ligation enzyme E3 (Wilkinson and Henley, 2010). The reversible deSUMOylation enzyme named the SUMO-specific protease can deconjugate SUMO from substrates in a process similar to that of the deubiquitylation process (Kim and Baek, 2009). In addition, a recent study has shown SUMOylation of DUBs usually inhibits its activity.

USP25, a member of the USP subfamily of DUBs, has been reported to be associated with the immune response, cancer, and other diseases (Li et al., 2014; Zhong et al., 2012). The N-terminus of USP25 contains two tandem UIM domains, and SIM and UBA domains (Denuc et al., 2009; Meulmeester et al., 2008). The target proteins of PTMs such as ubiquitination, phosphorylation, and SUMOylation are varied (Hecker et al., 2006; Song et al., 2004) (Figure 3A). SUMOylation at residues Lys99 and Lys141 of the UIM domain can inactivate USP25 and impair the ability of USP25 to hydrolyse the Ub chain by inhibiting the binding of USP25 to Ub chains *in vitro* (Figure 3C). Conversely, removing the SUMO modification from USP25 can increase its binding to the tetra-Ub chains (Meulmeester et al., 2008).

Coincidentally, USP28 is homologous to USP25 and is upregulated in colon cancer cells and NSCLC cells (Popov et al., 2007a; Valero et al., 2001; Li et al., 2014; Zhang L. et al., 2015). It also contains one UBA and two UIM domains at the N terminus that are responsible for the addition of SUMO (Zhen et al., 2014). SUMOylation at the Lys99 residue of the UIM domain could inhibit the activity of USP28 (Figure 3C), indicating its potential role in cancer therapy (Diefenbacher et al., 2014; Zhen et al., 2014; Zhang Y. et al., 2015).

CYLD, belonging to the USP subfamily, is ubiquitously expressed and highly conserved, and negatively regulates NF-κB activation by TNFR family members (Trompouki et al., 2003). The SUMOylation of CYLD at the Lys40 residue of its N-terminus can also reduce its activity against substrates TRAF2 and TRAF6 (Figure 3C), and block the activation of NF-kB signalling, which plays an essential role in inflammatory reactions (Kobayashi et al., 2015).

Studies have also shown that USP39 plays a vital role in cancer development, including breast cancer and hepatocellular cancer, where upregulation of USP39 was observed (Liu S. et al., 2015; Pan et al., 2015). Furthermore, USP39 can also undergo SUMOylation at several sites, including Lys6, Lys16, Lys29, Lys51, and Lys73 residues (Figure 3C). Inhibition of SUMOylation of USP39 enhanced the proliferation of cancer cells as it affected the recruitment of tri-snRNP, suggesting that SUMOylation of USP39 plays an essential role in cancer therapy (Wen et al., 2014; Wen et al., 2014).

The MJD subfamily member Ataxin-3 has been shown to undergo SUMOylation at its N-terminal Lys166 residue. SUMOylation does not alter subcellular localization, but promotes apoptosis (Shen L. et al., 2005; Guzzo and Matunis, 2013). Furthermore, the SUMOylation of Lys356 can influence the interaction between Ataxin-3 and p97, which implies its potential role in tumours (Ge et al., 2015). However, the precise mechanisms of this process are still unknown (Dantuma and Hoppe, 2012; Zhou YF. et al., 2013; Almeida et al., 2015).

### Oxidation Inhibits the Catalytic Activity of DUBs

Reactive oxygen species (ROS) and by-products of mitochondrial oxidative metabolism are continuously produced in eukaryotic cells (Poyton et al., 2009). Deregulated ROS levels are linked to various human diseases, including cancer, Alzheimer’s disease, and aging (Benhar et al., 2002; Butterfield and Boyd-Kimball, 2004; Hekimi et al., 2011). As most members of DUB families are reduced cysteine proteases, it is reasonable that DUBs can be regulated by ROS. Studies have shown that many members of the OTU, UCH and USP subfamily of DUBs can be reversibly inactivated by oxidation (Lee et al., 2013). Until now, researchers have purified about 30% of known DUBs, including members of the OTU, UCH and USP subfamilies, and most of them did not show any significant activity due to oxidation during purification. However, after DTT treatment, the activity of the DUBs were enhanced, indicating that oxidation can inhibit the catalytic activity of DUBs.

For example, purified USP19 exhibits a deubiquitinase weak activity. However, it can be activated by DTT treatment under reducing conditions and exhibits the ability to cleave K48-diUb or Ub-AFC (Mei et al., 2011). Nevertheless, when treated with H_2_O_2,_ the deubiquitinase activity was completely abolished as oxidation occurs on cysteine residues in the catalytic domain (Figure 3D). At the same time, this inactivation process can be reversed by addition of DTT (Lee et al., 2013).

Furthermore, some purified DUBs such as USP7, CYLD, and UCHL1 are active, but their activities can be further enhanced by adding DTT (Figure 3D). In contrast to USP19, USP7 and UCHL1 were more resistant to inhibition by H_2_O_2_ at pH 7.4, which indicates that the sensitivity of DUBs to oxidative inhibition depends on the original activity during which the deprotonation of cysteine in the catalytic domain occurs (Lee et al., 2013).

Notably, there are some purified DUBs that have no detectable activity with or without DTT, such as USP1, USP14, and USP28. These DUBs may require certain cofactors for their activity (Cohn et al., 2007). As for USP1, its activation requires the cofactor UAF1. The purified UAF1-USP1 complex was indeed active in the Ub-AFC assay, and the activity was strongly and reversibly inhibited by H_2_O_2_. Interestingly, the interaction of USP1 and UAF1 was not affected by H_2_O_2_, suggesting that the inhibition is likely caused by oxidation of the catalytic active site of USP1, which is consistent with other DUBs (Cotto-Rios et al., 2012; Huang et al., 2012; Lee et al., 2013).

Similar to that of the USP family, reversible oxidation can also occur on the members of the OTU subfamily of DUBs, such as OTUD7B, OTUD1, OTUD2, OTUD3, OTUD5, and OTUD6B. This indicates that it is a common regulatory mechanism of deubiquitinase activity (Kulathu et al., 2013).

## The Therapeutic Potential of Targeting PTMs in DUBs

Many DUBs undergo one or more PTMs, including phosphorylation, ubiquitination, SUMOylation, and oxidation, leading to changes in activation, inhibition, stability, or localization of these DUBs. These PTMs play a crucial role in the regulation of DUB and dysregulation of this process is associated with many diseases, including cancer, DDR, inflammatory, and neurodegenerative diseases (Table 3).

**TABLE 3:** PTMs of DUBs induced cellular effects and disorders.

Disorders	DUBs	PTMs	Cellular effects	Disorder type	References
*Cancer*	USP4	Phosphorylation (tumor promoter)	PI (3)K–AKT; enhanced TGF-β- signaling	Breast cancer	Zhang et al. (2012)
	USP10	Phosphorylation (tumor suppressor)	ATM-Mdm2; down-regulated p53 signaling	Tumor without mutation of p53	Yuan et al. (2010)
	OTULIN	Phosphorylation (tumor promoter)	ABL1/c-Abl; increased OTULIN/β-catenin interaction; activation of wnt/β-catenin signaling	Triple-negative breast cancer	Wang et al. (2020b); Wang and Wu (2020)
	USP13	Phosphorylation Ser114 (tumor suppressor)	Aurora B phosphorylates USP13 and promotes the interaction between USP13 and aurora B. USP13, in turn, can deubiquitinate aurora B, proper regulation of aurora B on cell cycle	Cancers where aurora B overexpression	Esposito et al. (2020)
	USP13	Phosphorylation Thr196 (tumor promoter)	ATM induced phosphorylation form of USP13 can deubiquitinate RAP80 and prompt DNA damage repair response	Ovarian cancer	Li et al. (2017)
	USP13	Phosphorylation Tyr708 (tumor promoter)	Phosphorylation of USP13 at Tyr708 induced by CLK3 promotes the cholangiocarcinoma progression by activating c-Myc mediated purine synthesis	Cholangiocarcinoma with CLK3 mutation	Zhou et al. (2020)
	CYLD	Phosphorylation (tumor promoter)	IKKε-induced phosphorylation of CYLD decreased the activity of it and completely blocks the CYLD-mediated deubiquitination of TRAF2, thereby promoting the transformation and progression of breast cancer cell	Breast cancer	Hutti et al. (2009)
	USP28	SUMOylation (tumor suppressor)	SUMOylation at Lys99 residues of USP28 could inhibit the activity of USP28	Colon cancer cells and NSCLC cells	Masoumi et al. (2016)
	USP39	SUMOylation (tumor suppressor)	Inhibition of the SUMOylation of USP39 can enhance the proliferation of cancer cells *via* affecting the recruitment of tri-snRNP	Breast cancer and hepatocellular cancer	Masoumi et al. (2016); Liu et al. (2015b); Pan et al. (2015); Wen et al. (2014)
	USP14	Unknown	Protein turnover	Ovarian and lung cancer	Wang et al. (2015a); Wu et al. (2013)
	UCHL5	Unknown	Protein turnover	Esophageal and ovarian cancer	Chen et al. (2012); Wang et al. (2014)
	USP11	Unknown	Unknown	Breast cancer	Bayraktar et al. (2013)
	USP8	Unknown	Regulation of the recycle of receptor tyrosine kinases, such as EGFR	Lung cancer	Reincke et al. (2015)
	UCH37	Unknown	Unknown	Carcinoma	Chen et al. (2012)
	USP15	Unknown	Regulation of the TGFβ signaling pathway	Breast cancer, ovarian cancer, glioblastoma	Eichhorn et al. (2012); Inui et al. (2011)
DNA damage response	USP7	Phosphorylation (DNA repair promoter)	CK2-Mdm2; down-regulated p53 signalling	DNA damage response	Khoronenkova et al. (2012)
	USP4	Auto-deubiquitination (DNA repair promoter)	Auto-deubiquitination is required for USP4 to interact with CtIP/MRN and promote DNA repair	DNA repair	Wijnhoven et al. (2015)
	USP1	Phosphorylation (DNA repair promoter)	After USP1 is phosphorylated, the USP1/UAF complex translocated to the nucleus and recruit FANCD2/PCNA substrates to regulate DNA damage response	Tanslesion DNA repair	Garcia-Santisteban et al. (2012); Olazabal-Herrero et al. (2015)
	UCHL3	Phosphorylation (DNA repair promoter)	ATM-induced phosphorylation form of UCHL3 deubiquitinates RAD51 and promote its binding to BRCA2 after DNA damage	DNA damage repair and resistance of cancer cell to chemotherapy	Luo et al. (2016)
	USP11	Unknown	Targeted PALB2	Homologous recombination	Bayraktar et al. (2013)
	USP9X	Unknown	Targeted claspin	Replication checkpoint	Murtaza et al. (2015)
Inflammatory	A20	Phosphorylation (positive regulator)	IκKβ-mediated phosphorylation of A20 at residue Ser381 facilitate A20 to cleave K63-linked polyubiquitin chains	Suppress inflammatory signalling	Wertz et al. (2015)
	USP9X	Phosphorylation (positive regulator)	TCR-dependent phosphorylation of USP9X enhances its catalytic activity and deubiquitinate ZAP70	T Lymphocyte activation	Naik and Dixit (2016)
	OTUD4	Phosphorylation (positive regulator)	CKII-induced phosphorylation of OTUD4 promote it to hydrolyze the ubiquitin chain changed from K48 to K63, playing an essential role in innate immune signalling	Innate immune signalling	Zhao et al. (2018)
	CYLD	SUMOylation (positive regulator)	SUMOylation of CYLD at Lys40 can reduce its activity and block the activation of NF-kB signalling	Inflammatory	Masoumi et al. (2016)
	OTULIN	Unknown	Targeted on NEMO and RIPK1/2	Inhibit NF-κB signaling	Iwai et al. (2014)
	USP18	Unknown	Expression regulated by IFNγ	Function in haematopoietic cell differentiation	Malakhov et al. (2002) Malakhova et al. (2006)
	USP25	Unknown	Expression regulated by IRF7 and IFN	Regulation of innate immune response to DNA and RNA virus	Lin et al. (2015)
	USP7	Unknown	Negative regulator of NF-κB activity	T_reg_ response	Colleran et al. (2013)
	USP21	Unknown	Stabilize FOXP3	T_reg_ response	Zhang et al. (2013)
	Cezanne	Unknown	Positive regulation of T cell receptor signalling and deubiquitinate ZAP70	T_H_1 and T_H_17 response	Hu et al. (2016)
	TRABID	Unknown	Targeted JMJD2D	Positive regulator of IL-22 and IL-23 production	Jin et al. (2016)
	USP4	Unknown	Targeted TAK1 to downregulate NF-κB activation	Highly expressed in CD4^+^ T cells form rheumatic heart disease	Wang et al. (2013)
	USP10	Unknown	Stabilize T-bet	Highly expressed in PBMCs from patients with asthma	Pan et al. (2014)
	USP17	Unknown	Regulation the stability of IL-33	T_H_1 and T_H_17 response	Chen et al. (2010); Ni et al. (2015)
	USP18	Unknown	Regulate TAK1-TAB1 interaction	Expression induced by cytokines	Liu et al. (2013)
Neurodegenerative diseases	USP25	Phosphorylation (promoter)	VRK2 kinase-mediated phosphorylation of USP25 suppresses the deubiquitinating activity of USP25 and stabilize the molecular chaperone TRiC protein	Misfolded protein aggregation in neurodegenerative disease	Kim et al. (2015)
	Ataxin3	Ubiquitination (suppressor)	Ubiquitination of Ataxin3 can enhance its ubiquitin chain cleavage activity and improve protein quality control	Closely related to the neurodegenerative disorder	Todi et al. (2010)
	Ataxin3	Phosphorylation (suppressor)	Phosphorylation of Ataxin3 can regulate its aggregation and counter the neuromorphologic defects by decreasing its deubiquitinase activity	Machado-joseph disease	Fei et al. (2007; Matos et al. (2016)
	UCHL1	Ubiquitination (promoter)	Monoubiquitination of the residues near the active site of UCHL1 can restrict its enzymatic activity by inhibiting the binding of ubiquitin to UCHL1	Neurodegenerative diseases, including Alzheimer’s and Parkinson’s disease	Meray and Lansbury (2007)
	USP7	Unknown	Antagonizes ubiquitination of α-Synuclein and regulation of REST signaling and neuronal differentiation	Neurodegeneration disease	Alexopoulou et al. (2016)
	USP8	Unknown	Regulates mitophagy by cleaving ubiquitin from parkin	Neurodegeneration disease	Durcan et al. (2014)
	USP14	Unknown	Promotes the clearance of tau or Ataxin3 protein involved in neurodegeneration	Mutation lead ataxia	Wilson et al. (2002)
	USP15	Unknown	Counteract parkin-mediated mitophagy	Glioblastoma	Cornelissen et al. (2014)
	USP30	Unknown	Dysfunction of mitochondrial	Neurodegeneration	Bingol et al. (2014)

### PTMs of DUBs Are Closely Related to Cancers

PTMs in DUBs can promote cancer progression. A study demonstrated that AKT-mediated phosphorylation of USP4 was associated with poor prognosis in breast cancer patients. A building crosstalk between TGF-β and AKT signalling pathways exists, which indicates a potential therapeutic role (Zhang et al., 2012). Additionally, DNA damage-induced ABL1/c-Abl (ABL proto-oncogene 1) activation can promote the phosphorylation of OTULIN, which enhances its interaction with β-catenin and promotes the activation of Wnt/β-catenin signalling (Table 3) This mechanism plays a critical role in the triple-negative breast cancer (TNBC) progression, metastasis, and drug resistance to cancer treatments (Douglas and Saleh, 2019; Wang Y. et al., 2020; Wang and Wu, 2020).

Furthermore, CLK3-induced phosphorylation of the Tyr708 residue in USP13 promotes cholangiocarcinoma progression by activating c-Myc-mediated purine synthesis (Zhou et al., 2020). Moreover, ATM-induced phosphorylation of the Thr196 residue in USP13 after DNA damage functions as an essential regulatory event, and plays a critical role in the resistance of cancer cells to chemotherapy by deubiquitinating RAP80, promoting recruitment of complexes, and eliciting a DDR (Hu et al., 2011; Li et al., 2017) (Table 3).

Additionally, in primary adult T-cell leukemia/lymphoma (ATLL) samples and cell lines, increased IKK-induced CYLD phosphorylation was observed. Both IKK inhibitors and overexpression of kinase-inactive TBK/IKK can lower CYLD phosphorylation and trigger cell death (Table 3). These results indicated that phosphorylated CYLD is a crucial regulator of ATLL survival and a potential novel therapeutic target for pharmacologic modification in ATLL (Xu et al., 2020).

In contrast, PTMs of some DUBs act as tumour suppressors. For example, phosphorylated USP10 can deubiquitinate and stabilize p53 by reversing its nuclear export and Mdm2-induced degradation. Therefore, phosphorylated USP10 can inhibit the growth of tumour cells without inducing mutations in p53 (Table 3), which implies that phosphorylation of USP10 has potential therapeutic effects in tumours. Additionally, kinase Aurora B induced phosphorylation of the Ser114 residue in USP13 prevents several major human cancers by promoting its interaction with partners and stability (Esposito et al., 2020). A study showed that SUMOylation at Lys99 residues of USP28 could inhibit the activity of USP28 (Table 3), indicating potential therapeutic role in colon cancer cells and NSCLC cells (Diefenbacher et al., 2014; Zhen et al., 2014; Zhang L. et al., 2015). Furthermore, inhibition of the SUMOylation of USP39 can enhance the proliferation of cancer cells including breast and hepatocellular cancer *via* affecting the recruitment of tri-snRNP, suggesting that SUMOylation of USP39 has an essential role in cancer therapy (Wen et al., 2014; Liu H. et al., 2015; Pan et al., 2015) (Table 3).

Additionally, there are many other DUBs which are closely related to cancer and other diseases (Li et al., 2002; Xu et al., 2014; Yuan et al., 2015; Harrigan et al., 2018). For instance, dysregulation of USP14 can lead ovarian and lung cancer (Mines et al., 2009; Wu et al., 2013; Wang Y. et al., 2015), and abnormal of UCHL5 can also cause ovarian and esophageal cancer (Chen et al., 2012; Wang et al., 2014). Study found dysregulation of USP11 is related to breast cancer, but the mechanism underlying that is not clear (Bayraktar et al., 2013). Also, dysregulation of the recycle of tyrosine kinase like epidermal growth factor receptor (EGFR) by USP8 cause the development of lung cancer (Reincke et al., 2015). Moreover, abnormal regulation of the TGFβ signaling pathway by USP15 can lead the occurrence of glioblastoma, breast cancer, and ovarian cancer (Inui et al., 2011; Eichhorn et al., 2012; Hayes et al., 2012). In addition, the expression of UCH37 is closely related to the progression of human esophageal squamous cell carcinoma (Chen et al., 2012). However, if they are PTMs-mediated disorders or not and which kind of PTMs mediated the disorder was still not clear. Therefore, further investigations need to be done to clarify the corelation between DUBs-related disorders and PTMs.

### PTMs of DUBs Promote DNA Repair

PTMs of DUBs plays vital roles in DNA repair after exposure to genotoxic agents or chemotherapy. Studies have shown that USP7 phosphorylation promotes the deubiquitination and stabilization of Mdm2, which in turn leads to the degradation and downregulation of p53 (Table 3) (Khoronenkova and Dianov, 2012; Khoronenkova et al., 2012). Also, the auto-deubiquitination of USP4 is required for USP4 to interact with CtIP/MRN and promote DNA repair (Wada and Kamitani, 2006; Wijnhoven et al., 2015). Additionally, ATM-induced phosphorylation of UCHL3 occurs after DNA damage, which in turn deubiquitinates RAD51 and promotes the binding between BRCA2 and RAD51 (Table 3). Thus, these processes play an important role in DNA damage repair and the resistance of cancer cells to radiation and chemotherapy (Adelina A. et al., 2001; Luo et al., 2016; Zhao et al., 2017). There are some other DUBs such as USP11, USP28 and USP9X were reported to be closely related to DNA repair, but if it is the PTMs induced or not was not clear until now (Zhang et al., 2006; Wiltshire et al., 2010; Bayraktar et al., 2013; Homan et al., 2014; Murtaza et al., 2015).

### PTMs of DUBs Positively Regulate Inflammation

PTMs of DUBs can positively regulate the function of DUB in the process of inflammation. Firstly, IκKβ-mediated phosphorylation of A20 at residue Ser381 plays an essential role in facilitating cleavage of K63-linked polyubiquitin chains by A20, indicating the potential role of A20 in suppressing inflammation-associated signalling (Song et al., 1996; Wertz et al., 2015) (Table 3). In addition, TCR-dependent phosphorylation of USP9X enhances its catalytic activity and makes it competent to deubiquitinate ZAP70, which functions as a positive regulatory switch during T lymphocyte activation by removing the monoubiquitination inhibition of ZAP70 (Naik and Dixit, 2016) (Table 3). Furthermore, CKII-induced phosphorylation of OTUD4 promoted the preferential hydrolysis of the Ub chain changed from K48- to K63-linked chains, which plays an essential role in innate immune signalling (Zhao et al., 2018). Similarly, the SUMOylation of CYLD at Lys40 residue can also reduce its activity and block the activation of NF-kB signalling, playing an essential role in inflammation (Kobayashi et al., 2015) (Table 3).

There are many other DUBs have been demonstrated to have important role in the inflammation. However, whether PTMs of DUBs occurs and promote its involvement in inflammation response were unclear (Zhang et al., 2018). For example, OTULIN can inhibit the NF-κB signaling by targeted on NEMO and the receptor-interacting protein kinase 1/2 (RIPK1/2) (Iwai et al., 2014; Damgaard et al., 2016). And the expression of USP18 was regulated by interferon gamma (IFNγ) and play an important role in haematopoietic cell differentiation (Malakhov et al., 2002). Meanwhile, expression of USP25 was regulated by interferon regulatory factor 7 (IRF7) and IFN, and have critical role in the innate immune response to DNA and RNA virus (Lin et al., 2015). USP7 and USP21 play an important role in regulatory T cell (T_reg_) response by negatively regulate NF-κB activity and stabilize the forkhead box p3 (FOXP3) respectively (Colleran et al., 2013; Zhang et al., 2013). Moreover, Cezanne can positively regulate T cell receptor signaling, playing important role in T helper cell 1 (T_H_1) and T_H_17 response (Pareja et al., 2012; Hu et al., 2016). TRABID can positively regulate the production of interleukin 22 (IL-22) and IL-23 by targeting on JMJD2D (Jin et al., 2016). Furthermore, USP4 targeted on TGFβ-activated kinase 1 (TAK1) to downregulate NF-κB activation, which was highly expressed in CD4^+^ T cell from rheumatic heart disease (Wang et al., 2013). Similarly, USP10 can stabilize T-bet and highly expressed in the peripheral blood mononuclear cells (PBMCs) from patients with asthma (Pan et al., 2014). USP17 play important role in T_H_1 and T_H_17 response by stabilizing IL-33 (Burrows et al., 2009; Chen et al., 2010; Ni et al., 2015). USP18 can regulate TAK1-TAB1 interaction, and the expression was induced by cytokines (Burkart et al., 2012; Liu et al., 2013). Therefore, DUBs were closely involved in the inflammatory signaling pathway and inflammatory disease. However, if PTMs promote and take part in this process or not still need further study in the future.

### PTMs of DUBs Is Closely Related to Neurodegenerative Diseases

Dysregulation of DUBs can cause various neurodegenerative diseases, including Alzheimer’s and Parkinson’s disease (Harrigan et al., 2018). Studies demonstrated that PTMs of DUBs can either promote or suppress the progression of neurodegenerative diseases. VRK2 kinase-mediated phosphorylation of USP25 suppressed the deubiquitinating activity of USP25 and stabilized the molecular chaperone TRiC (Table 3), leading to the aggregation of misfolded proteins in neurodegenerative diseases (Kim et al., 2014; Kim et al., 2015). Studies showed that the ubiquitination of Lys117 near the catalytic groove of Ataxin3 can enhance its Ub chain cleavage activity and this activity is closely related to neurodegenerative disorders (Todi et al., 2009; Todi et al., 2010) (Table 3). Additionally, studies demonstrated that phosphorylation of Ataxin3 influences its aggregation and counters the neuromorphologic defects occurring due to it by decreasing its deubiquitinase activity (Lim et al., 2006; Scaglione et al., 2013). This mechanism plays an important role in the development of Machado-Joseph disease (Fei et al., 2007; Matos et al., 2016) (Table 3). Monoubiquitination of the residues near the active site of UCHL1 can restrict its enzymatic activity by inhibiting the binding of Ub to UCHL1 (Table 3), promoting the progression of neurodegenerative diseases, including Alzheimer’s and Parkinson’s disease (Das et al., 2006; Meray and Lansbury, 2007; Liu et al., 2009).

Additionally, there are many other DUBs which can lead neurodegenerative diseases. For example, study showed that USP7 is also involved in the neurodegeneration through antagonize the ubiquitination of α-synuclein and regulate the RE1 silencing transcription factor (REST) signaling pathway (Alexopoulou et al., 2016). Similarly, USP8 is closely related to the development of neurodegeneration *via* regulating the mitophagy by cleaving the ubiquitin from Parkin (Durcan et al., 2014; Alexopoulou et al., 2016). USP14 can promote the clearance of Tau or Ataxin3 protein which is aggregated in neurodegeneration (Wilson et al., 2002; Homma et al., 2015). Moreover, USP15 plays an important role in glioblastoma by counteracting Parkin-mediated mitophagy (Cornelissen et al., 2014). Study also showed that USP30 is involved in the neurodegenerative disease by leading mitochondrial dysfunction (Bingol et al., 2014). However, whether the regulation effect of PTMs on these DUBs is the reason that lead the occurrence of DUBs-related neurodegenerative diseases still need further study.

## Discussion

Generally, single PTMs regulate the function of DUBs *via* allosteric regulatory effects that lead to conformation changes or by exposing a new binding site by abolishing original protein-protein interactions (Figure 4D). For example, phosphorylation of USP4 is essential for protein stability, or for forming a complex with itself or another protein partner such as USP15. Meanwhile, phosphorylation can prevent USP4 from being localized in the nucleus and play a vital role in DDR.

Moreover, complex crosstalk between post-translationally modified proteins often occurs; a well-described example are kinases. Under certain conditions, phosphorylation is often necessary to trigger subsequent phosphorylation, ubiquitination, or SUMOylation, and the ubiquitination or methylation of histones may be essential for its acetylation (Hunter, 2007). The crosstalk of post-translationally modified DUBs can be either positive or negative. For instance, the N-terminal of USP25 is a target of a variety of PTMs, including phosphorylation, ubiquitination, SUMOylation, and acetylation (Figure 3A). Phosphorylation of USP25 can decrease its protein level in a proteasomal degradation-independent manner by inhibiting the ubiquitination of USP25. Ubiquitination and SUMOylation occur at the same Lys99 residue of USP25. In addition, ubiquitination and SUMOylation can occur at the same Lys99 residue of USP25. Therefore, there is a potential for opposing functions of activation and inhibition due to the negative crosstalk that might exist between ubiquitination and SUMOylation. At the same time, phosphorylation can promote the interaction of USP1 and its cofactor UAF1, whereas the binding of OTULIN to LUBAC is blocked by phosphorylation.

Recent literature clearly highlights the importance of PTMs in modifying the function of DUBs, and its role in promoting or decreasing the occurrence of diseases. PTMs are therefore emerging as a pivotal regulator of DUBs and may provide novel insights toward the biological functions of DUBs. However, the biological role of PTMs on DUBs has not been fully understood yet, and this requires further research. Structural studies will be particularly important in elucidating the biological role of PTMs in DUBs. There is no doubt that these studies will drive DUB-targeted drug discovery (Harrigan et al., 2018).

Recently, several small molecule inhibitors targeted toward different members of DUB subfamilies have been reported. Until now, there are few target-specific inhibitors that have been found (Ritorto et al., 2014). In the last two years, several highly specific inhibitors of USP7 and USP14 have been reported (Lee et al., 2010; Turnbull et al., 2017; Gavory et al., 2018; Wang et al., 2018; Clague et al., 2019). However, as many DUB members were strictly conserved during evolution and have a high sequence similarity between each other, new perspectives are still required to facilitate the development of selective compounds targeted toward DUB. Subsequently, novel insights into the PTMs-mediated regulation of the function of DUBs might provide us opportunities to develop efficient drugs targeting DUBs. Combining inhibitors of DUBs and enzymes responsible for regulatory PTMs, such as kinases or phosphatase inhibitors, will provide more efficient entry points for pharmacological intervention strategies. For instance, drugs targeting proteins of the Ub/proteasome and SUMO pathways, such as DUBs and SUMO metabolism enzymes, are either on the way or have already entered clinical trials for cancer therapy (Masoumi et al., 2016). A better understanding of the cross-talk or interplay between these two pathways can lead to the identification of novel anticancer tools for treating diseases in which SUMOylation plays a significant role.

Moreover, several DUBs have been reported to be overexpressed or mutated in cancer resulting in altered activities. As PTMs can regulate the abundance and activity of DUBs, it may serve as an effective target for novel cancer therapeutic approaches. We anticipate that the outcome of DUB-focused regulatory research will help decipher the molecular basis of the pathogenesis of human disorders and thus lead to novel or improved therapeutic strategies. We hope that the paradigms presented in this commentary of how diversification and regulation of PTMs in DUBs are achieved will guide future research.
